# Improving phloroglucinol tolerance and production in *Escherichia coli* by GroESL overexpression

**DOI:** 10.1186/s12934-017-0839-x

**Published:** 2017-12-19

**Authors:** Rubing Zhang, Yujin Cao, Wei Liu, Mo Xian, Huizhou Liu

**Affiliations:** 1 CAS Key Laboratory of Bio-Based Materials, Qingdao Institute of Bioenergy and Bioprocess Technology, Chinese Academy of Sciences, Qingdao, 266101 China; 2 University of Chinese Academy of Sciences, Beijing, 100049 China

**Keywords:** GroESL, Expression level, Tolerance, *Escherichia coli*, Phloroglucinol

## Abstract

**Background:**

Phloroglucinol is an important chemical which has been successfully produced by engineered *Escherichia coli*. However, the toxicity of phloroglucinol can enormously inhibit *E. coli* cell growth and viability, and the productivity is still too low and not economically feasible for industrial applications. Therefore, strain tolerance to toxic metabolites remains a key issue during the production of chemicals using biological processes.

**Results:**

In the present work, we examined the impact of the native GroESL chaperone system with different overexpression levels on phloroglucinol tolerance and production in *E. coli*. The *groESL* gene was cloned into an expression vector, of which expression level was regulated by three different promoters (natural, tac and T7 promoter). Strain tolerance was evaluated employing viable cell counts and phloroglucinol production. In comparison with the control strain, all GroESL overexpressing strains showed good characteristics in cell viability and phloroglucinol synthesis. Strain which overexpressed GroESL under tac promoter was found to show the best tolerance in all of those tested, resulting in a 3.19-fold increase in viable cell numbers compared with control strain of agar-plate culture under the condition of 0.7 g/L phloroglucinol, and a 39.5% increase in phloroglucinol production under fed-batch fermentation. This engineered strain finally accumulated phloroglucinol up to 5.3 g/L in the fed-batch cultivation 10 h after induction, and the productivity was 0.53 g/L/h. To date, the highest phloroglucinol production was achieved in this work compared with the previous reports, which is promising to make the bioprocess feasible from the economical point.

**Conclusions:**

The data show that appropriate expression level of GroESL plays a critical role in improving phloroglucinol tolerance and production in *E. coli*, and maybe involve in controlling some aspects of the stress response system through upregulation of GroESL. GroESL overexpression is therefore a feasible and efficient approach for improvement of *E. coli* tolerance.

**Electronic supplementary material:**

The online version of this article (10.1186/s12934-017-0839-x) contains supplementary material, which is available to authorized users.

## Background

Phloroglucinol and its derivatives have been widely used in pharmaceuticals, leather industry and dyeing industry. For example, phloroglucinol as a precursor for different derivatives synthesis can be used as sedative, cosmetic additive and explosive [1]. Specifically, phloroglucinol shows antimicrobial activity against oral bacteria [2]. At present, phloroglucinol is mainly produced by chemical synthesis, of which the widespread utilization is limited by several problems such as safety, pollution and efficiency [3]. Currently, phloroglucinol synthesis using biotechnological method has attracted more attentions. The use of *Pseudomonas* spp. for producing phloroglucinol derivative has been investigated by many researchers [4, 5]. *Arabidopsis* plants were also employed to produce phloroglucinol by expressing a bacterial gene *phlD* [3]. Moreover, the engineered *Escherichia coli* had been used to produce phloroglucinol and its derivatives from renewable feedstock [6, 7]. It is environmentally safe to synthesize phloroglucinol using biotechnological methods. However, the microbial production of phloroglucinol is still challenging, the highest production titer reported was only 3.8 g/L under fed-batch fermentation conditions using recombinant *E. coli* and was too low to be applied in industrial production [7]. The inherent toxicity of phloroglucinol to bacteria is a major limiting factor for the production titers during fermentation or whole-cell biocatalysis [8]. The study shows that cell growth of *E. coli* can be significantly inhibited, when phloroglucinol concentration is more than 0.5 g/L in culture media [7].

In a previous study, the gene *marA* (multiple antibiotic resistances) which can regulate the response to multiple environmental stresses was employed to improve *E. coli* tolerance to phloroglucinol. Results showed that the gene *marA* overexpression could improve not only phloroglucinol tolerance but also phloroglucinol production in the recombinant *E. coli* [7]. Moreover, phloroglucinol was removed from fermentation broth through absorbent resin during fermentation process to alleviate the stress, and the final production was also improved [9]. Therefore, the toxicity of phloroglucinol compound prevents further increase in production titer. In order to obtain a higher titer for industrial applications, improving the phloroglucinol tolerance in *E. coli* strains is a promising strategy. Strain tolerance to metabolites is also an important issue for the microbial synthesis of many valuable chemicals.

The microbial tolerance to toxic chemicals is complex and affected by many parameters such as pH, temperature, osmotic pressure, etc. [10]. Several mechanisms of action work together to show tolerance, including cellular transport, changes in membrane properties, regulation of energy metabolism, etc. [11, 12]. Various kinds of genes or gene clusters dispersed on the chromosome or located on plasmids have been found to participate in resistance regulation.

The heat shock proteins (HSPs), also called molecular chaperones, play an essential role in the synthesis, folding and transport of proteins as well as remediation of damage to misfolded proteins [13]. Several studies have also shown that the HSPs can respond to many kinds of environmental stresses and play an important role in improving solvent, acid and heat tolerance [13–15]. Transcriptome analyses indicate that transcriptional level of HSPs gene would be upregulated, when *E. coli* strains were exposed to ethanol, *n*-butanol, *i*-butanol [16–18]. Moreover, HSPs also play an important role in solvent stress response in a variety of other organisms including *Streptococcus pneumoniae* and *Lactobacillus brevis* [19, 20]. In *E. coli*, three main molecular chaperone systems are involved in stress response, including trigger factor, GroEL–GroES, and DnaK–DnaJ–GrpE [21]. The GroESL is a member of Hsp60 chaperone family and can mediate protein transition between soluble and insoluble status [22], and the mechanism of GroESL system action has been elucidated [23]. It has been shown that overexpression of autologous and heterologous GroESL in *E. coli* could result in improving tolerance to a variety of solvents [23, 24]. Expressing the GroESL, the growth and survivability of *E. coli* have been greatly improved in the presence of different toxic alcohols [23]. The expression levels of GroESL were also regulated by the inducible promoters and plasmid copy number, and thus leading to the impact on microbial tolerance [15, 23]. Furthermore, GroESL overexpression could not only enhance tolerance but also result in increased final product titers 40% higher than the wild type strains [25]. Therefore, *groESL* genes seem to be able to up-regulate under toxic chemical stresses, and the GroESL would be quite beneficial for the production of variously toxic chemicals.

With the aim to improve phloroglucinol tolerance and production in engineered *E. coli*, we reconstructed the metabolically engineered strains by GroESL overexpression. Considering that GroESL overexpression could enhance tolerance to a variety of alcohols in previous work, we tried to examine if overexpressing the GroESL system under its natural promoter could improve phloroglucinol tolerance and production in *E. coli*. The expression levels of GroESL were further modulated by employing different promoters, and the possible impacts on phloroglucinol tolerance and production in engineered *E. coli* were investigated.

## Methods

### Medium and culture conditions

*Escherichia coli* strains were cultivated in liquid Lysogeny Broth (LB) broth or on LB agar plates for gene cloning and protein expression. For phloroglucinol production, recombinant strains were cultivated in shake-flask or fed-batch fermentation using the fermentation media containing 9.8 g/L K_2_HPO_4_, 3.0 g/L (NH_4_)_2_SO_4_, 2.1 g/L citric acid monohydrate, 0.3 g/L ferric ammonium citrate, 0.24 g/L MgSO_4_, and 20.0 g/L initial glucose as well as 1 mL of trace element solution which contained 0.37 g/L (NH_4_)_6_Mo_7_O_24_·4H_2_O, 0.29 g/L ZnSO_4_·7H_2_O, 2.47 g/L H_3_BO_4_, 0.25 g/L CuSO_4_·5H_2_O, and 1.58 g/L MnCl_2_·4H_2_O. Appropriate antibiotics (50 μg/mL of kanamycin, Kan or 34 μg/mL of chloramphenicol, Cm) were added to the culture media according to selectable marker gene of each plasmid listed in Table 1.

**Table 1:** Plasmids and strains used in this study

Plasmids and strains	Relevant characteristics	References
Plasmids		
pET-28a(+)	*oripBR322 lacI* ^*q*^ *T7p, Kan* ^*r*^	Novagen
pET-phlDmarA	pET30a carrying *phlD* from *P. fluorescens* Pf5, *marA* from *E. coli* DH5α, *Kan* ^*r*^	[7]
pA-accADBC	pACYCduet-1 carrying *ACCase* from *E. coli* DH5α, *Cm* ^*r*^	[7]
pA-phlD/marA/acc	pACYCDuet-1 carrying *phlD* from *P. fluorescens* Pf5, *marA* and *ACCase* from *E. coli* DH5α, *Cm* ^*r*^	This study
pET28a-groESL	pET-28a(+) carrying *groESL* from *E. coli* BL21(DE3), natural promoter, *Kan* ^*r*^	This study
pET28a-tac-groESL	pET-28a(+) carrying *groESL* from *E. coli* BL21(DE3), Tac promoter, *Kan* ^*r*^	This study
pET28a-T7-groESL	pET-28a(+) carrying *groESL* from *E. coli* BL21(DE3), T7 promoter, *Kan* ^*r*^	This study
Strains		
BL21(DE3)	F^−^ *omp* T, *hsd*S_B_ (r_B_^−^m_B_^−^), *gal*, *dcm me*131, λ(DE3)	Invitrogen
G0	*E. coli* BL21(DE3)/pET-28a(+)	This study
G1	*E. coli* BL21(DE3)/pET28a-groESL	This study
G2	*E. coli* BL21(DE3)/pET28a-tac-groESL	This study
G3	*E. coli* BL21(DE3)/pET28a-T7-groESL	This study
PG0	*E. coli* BL21(DE3)/pA-phlD/marA/acc, pET-28a(+)	This study
PG1	*E. coli* BL21(DE3)/pA-phlD/marA/acc, pET28a-groESL	This study
PG2	*E. coli* BL21(DE3)/pA-phlD/marA/acc, pET28a-tac-groESL	This study
PG3	*E. coli* BL21(DE3)/pA-phlD/marA/acc, pET28a-T7-groESL	This study

### Strains and plasmids

All strains and plasmids used in this study are showed in Table 1. Primers used in this study are synthesized by GeneWiz (Suzhou, China) and listed (Additional file 1: Table S1). *E. coli* DH5α and BL21(DE3) were used for all plasmid constructions and expression of recombinant proteins. The expression vector pET-28a(+) was purchased from Novagen (Madison, WI, USA). The genes of *phlD* and *marA* were amplified together from plasmid DNA of pET-phlDmarA using the primers phlDmarA_F and phlDmarA_R. Then the PCR products were digested with *Not*I and *Afl*II, and cloned into the corresponding sites of pA-accADBC to construct plasmid pA-phlD/marA/acc.

The operon (including natural promoter, operator gene and structural genes) of *groESL* was amplified from genomic DNA of *E. coli* BL21(DE3) with the primers groESL-F_BglII and groESL-R_EcoRI. The PCR product digested with *Bgl*II and *EcoR*I was cloned into pET-28a(+) cut with the same restriction enzymes, generating pET28a-groESL. The *groESL* genes coding DNA sequences (CDS) fragment was PCR amplified using the primers T7-groESL-F_NcoI and T7-groESL-R_EcoRI, and cloned into pET-28a(+) between *Nco*I and *EcoR*I sites, to generate plasmid pET28a-T7-groESL. The primers of tac-groESL-F_BglII and tac-groESL-R_XbaI form a double-stranded DNA using the following a modification of PCR procedures: 95 °C for 3 min, followed by 6 cycles of 95 °C for 30 s and 60 °C for 30 s. The double-stranded DNA was then cloned into pET28a-T7-groESL digested with *Bgl*II and *Xba*I, generating pET28a-tac-groESL.

### Protein expression analysis by SDS-PAGE

The expression of GroESL under different promoters in *E. coli* BL21(DE3) was analyzed by SDS–polyacrylamide gel electrophoresis (PAGE). The single colonies of *E. coli* strain grew on LB agar were chosen and inoculated in liquid LB seed medium containing appropriate antibiotics. Seed cultures grown overnight were used as 1% inoculum in 100 mL LB liquid medium. The cells were cultivated in medium until OD_600_ to 0.7 at 37 °C and then induced by 0.2 mM isopropyl-β-d-thiogalactopyranoside (IPTG) at 30 °C for 6 h. The equal amounts (volume × OD_600_) of recombinant *E. coli* cells were harvested by centrifugation at 4 °C, washed twice with PBS buffer (10 mM Na_2_HPO_4_, 2 mM KH_2_PO_4_, 137 mM NaCl, 2.7 mM KCl, pH 7.4) and then resuspended in the equal volume of PBS buffer. The cells were fully disrupted by sonication on ice. After centrifugation at 13,000*g* for 10 min at 4 °C, equal volume of supernatant from different engineered strain was analyzed by SDS-PAGE. G0 strain was used as the control.

### Effect of GroESL overexpression on strain growth

The phloroglucinol resistances of strains with different GroESL expression levels were determined by the plate method. *E. coli* BL21(DE3) harboring pET-28a(+), pET28a-groESL, pET28a-tac-groESL or pET28a-T7-groESL was grown in liquid LB media until OD_600_ to 0.7 at 37 °C and then induced by 0.2 mM IPTG for 2 h at 30 °C. The cells of each recombinant *E. coli* were serially diluted with fresh liquid LB, and the dilution level was determined by OD_600_ measurements. Then the equal amounts of cells were spread on LB plates containing 0.7 g/L phloroglucinol and 0.1 mM IPTG. The plates were incubated at 30 °C for 18–24 h, and then the plate count method was used to measure the number of colony forming units (CFUs) per milliliter culture to assess effective cells under phloroglucinol stress.

Next, the hypothesis that overexpression of GroESL under different promoters in *E. coli* BL21(DE3) would lead to different growth curves in the conditions of phloroglucinol stress was examined. Each strain was inoculated in liquid LB seed media, and then the equal amounts of overnight culture were inoculated in 100 mL LB liquid media. The cultures were incubated at 37 °C and 180 rpm until OD_600_ to 0.7, and then induced by 0.2 mM IPTG at 30 °C for 24 h supplemented with a final concentration of 0.7 g/L phloroglucinol. Culture samples were collected at regular intervals and cell density was measured by optical density at 600 nm.

### Shake-flask cultivation of the recombinant *E. coli* strains

Shake-flask experiments of phloroglucinol synthesis were performed in a series of 500 mL shake flasks containing 100 mL of fermentation media with Kan and Cm. The single clone grown on LB agar was chosen and inoculated in 20 mL LB seed media, and then seed cultures were used as 1% inoculum in 100 mL of fermentation media. The *E. coli* strains (PG0, PG1, PG2, PG3) harboring different recombinant plasmids were cultivated in the broth and incubated in a gyratory shaker at 37 °C and 180 rpm. When the OD_600_ of the culture reached about 0.7, a final concentration of 0.2 mM IPTG was added to the media. Then, the culture was further incubated at 30 °C for 12 h. Cell density and phloroglucinol production were measured during the whole fermentation courses. The whole experiment was performed in triplicate.

### Fed-batch fermentation for phloroglucinol biosynthesis

Fed-batch cultures were carried out in a 5-L glass fermenter (BIOSTAT B plus MO5L, Sartorius, Germany) containing 2 L fermentation media. 100 mL of inoculum was prepared by incubating the culture in shake flasks at 37 °C for 10 h. During the fermentation process, the temperature was first set at 37 °C and the pH was maintained at 7.0 by automated addition of ammonia. Antifoam (THIX 298, Yantai Hengxin Chemical Co., Ltd., China) was added to prohibit foam development by regulated system. The stirring speed was first set at 300 rpm and then associated with the dissolved oxygen (DO) to maintain about 20% saturation of DO. When the initial glucose was nearly exhausted, fed-batch mode was run by feeding a solution containing 700 g/L of glucose at appropriate rates and the residual glucose was maintained about 0.5 g/L. A final concentration of 0.2 mM IPTG was added to induce protein expression when the cell density (OD_600_) reached about 12 at 37 °C, and the culture temperature was switched to 30 °C. Fermentation parameters were the same as in shake-flask cultivation. Consumed glucose is calculated by the total and residual glucose.

### Analytical methods

Cell density was determined by measuring the absorbance at 600 nm (OD_600_) with a spectrophotometer (Cary 50 UV–vis, Varian). Samples were diluted in the appropriate medium to ensure an absorbance at 0.20–0.80.

During the fermentation processes, the concentration of residual glucose in shake flasks or fermenters was quantified using the SBA-40D biosensor analyzer (Biology Institute of Shandong Academy of Sciences, China).

The concentration of phloroglucinol was analyzed by high performance liquid chromatography (HPLC). Briefly, samples were centrifuged at 12,000*g* for 5 min, and then the supernatants were filtered by a 0.20 μm nitrocellulose filter and analyzed by a HPLC (Agilent 1200 series, USA) equipped with UV/vis. The column (C18, 5 µm, 250 × 4.6 mm, Agilent, USA) was eluted at 30 °C using acetonitrile/water (4/6, v/v) as the mobile phase at a flow rate of 1 mL/min. The detection wavelength was 254 nm.

## Results

### Plasmid construction and SDS-PAGE analysis

Based on our previous study [7], the plasmid pA-phlD/marA/acc was successfully constructed for phloroglucinol production with chloramphenicol resistance. Considering the conclusion from the previous study that expression of *groESL* could improve tolerance to various stresses [23, 26, 27], the potentiality of improving *E. coli* tolerance to phloroglucinol by the overexpression of *groESL* gene was investigated in this study. Although *E. coli* possesses a native *groESL* gene, the gene expression level is very low. Thus, the native *groESL* gene with its natural promoter was firstly amplified, which then was cloned into a high-copy number plasmid pET-28a(+) forming the plasmid pET28a-groESL under the natural promoter. The plasmids pET28a-T7-groESL and pET28a-tac-groESL were constructed under the T7 and tac promoters, respectively.

**Fig. 1:**
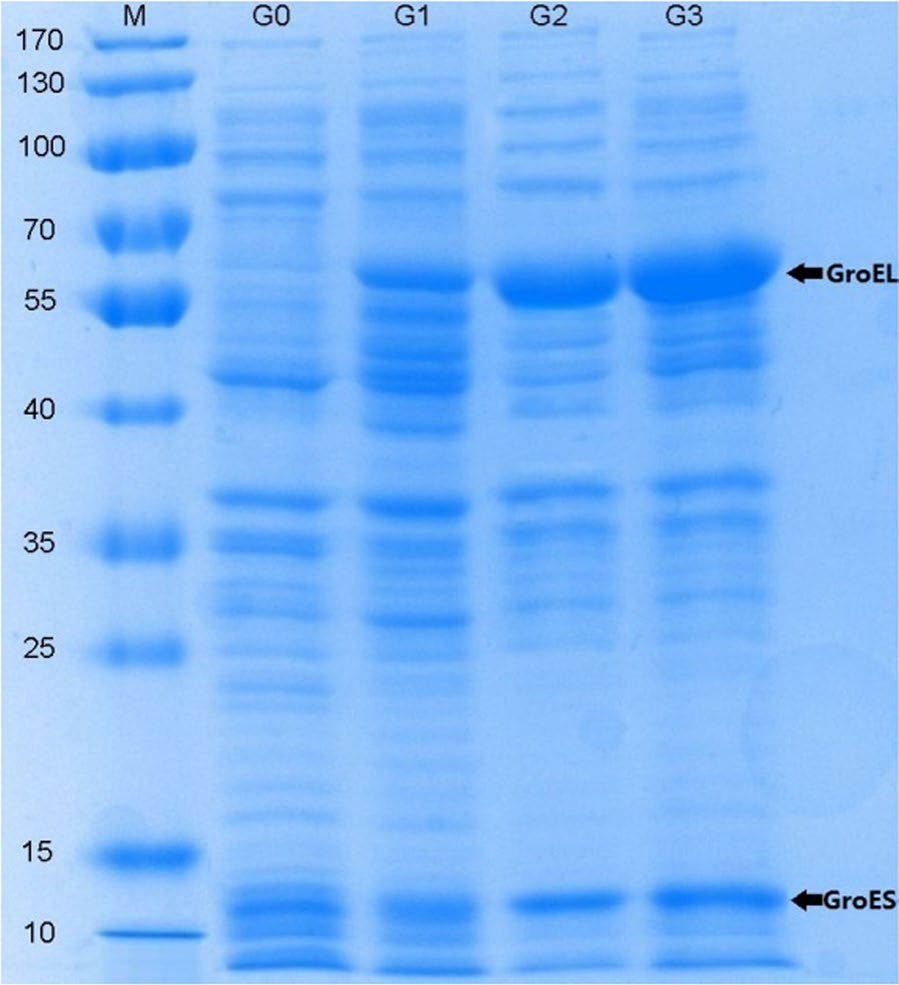
SDS-PAGE analysis of GroESL overexpression from different recombinant strains. Lane M unstained protein molecular weight marker (kD). The positions corresponding to the overexpressed GroES and GroEL proteins were indicated by an arrow

The expression levels of *groESL* under different promoter were verified as shown in Fig. 1. The SDS-PAGE patterns of samples from different recombinant strains analyzed with Coomassie brilliant blue staining showed that the recombinant proteins of GroESL were clearly overexpressed compared with the control strain. The target fragments of GroESL were consistent with the expected size, which were marked with arrows. Different expression levels of GroESL were observed under different promoters, and the GroESL expression levels gradually decreased with the changes of promoter from strong to weak. The strain G3 under the strong promoter T7 has the highest GroESL expression level, and the lowest GroESL expression level was found under its natural promoter. The expression of GroESL was not obvious in control strain G0. Our results indicated that the GroESL expression levels could be modulated by different promoters in *E. coli*.

### GroESL impact on *E. coli* tolerance to phloroglucinol

**Fig. 2:**
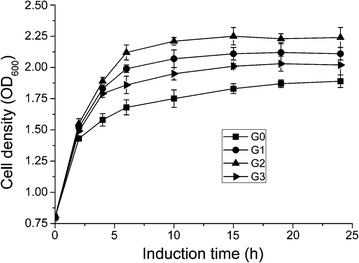
Cell growth of recombinant strains under phloroglucinol stress (0.7 g/L). The recombinant strains were cultured in liquid medium and induced with 0.2 mM IPTG at 30 °C. Control (G0, ■), GroESL overexpression with natural promoter (G1, ●), GroESL overexpression with tac promoter (G2, ▲), and GroESL overexpression with T7 promoter (G3, ▼). All experiments were done in triplet, and the error bars indicate standard deviations (n = 3)

The growth curves of different recombinant strains were measured under phloroglucinol stress as shown in Fig. 2. The optical density (OD_600_) revealed that overexpression of the *groESL* gene could lead to a faster growth compared with the control. Under phloroglucinol stress, a higher optical density and a longer exponential growth phase were observed in all GroESL overexpressing strains compared with the control. These results confirmed that the GroESL can enhance tolerance to phloroglucinol and prolong the exponential growth phase of recombinant *E. coli*.

**Fig. 3:**
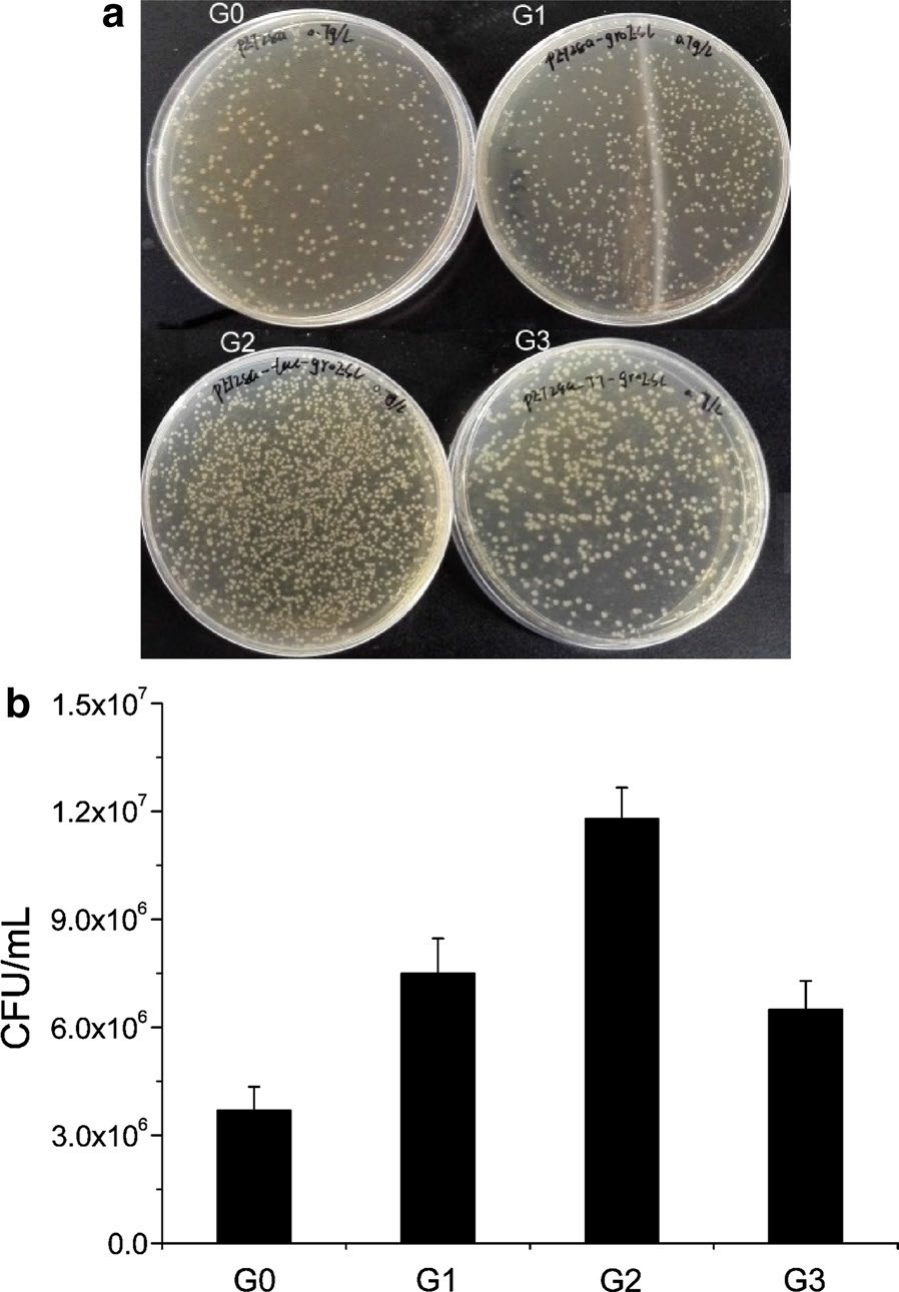
Tolerance assays for GroESL overexpression of *E. coli* BL21(DE3). **a** On LB agar plate. **b** Viable cell counts (CFU/mL). Strains were induced by 0.2 mM IPTG for 2 h, diluted and spread on LB agar plates under 0.7 g/L phloroglucinol and 0.1 mM IPTG, and then incubated at 30 °C for 20 h

Under the condition of phloroglucinol stress, the survivability of GroESL overexpressing strains was assessed by the plate count method. The results showed that more effective cells were obtained in GroESL overexpressing strains than control strains under phloroglucinol stress (Fig. 3a), and thus more effective cells could be involved in phloroglucinol synthesis in the GroESL overexpressing strains. The strain G2 (overexpression of GroESL under tac promoter) has the largest number of viable cells compared with other strains, and a 3.19-fold increase in the number of CFU/mL was measured compared with the control (Fig. 3b). Although GroESL overexpressing strain G1 and G3 produce a 2.03- and 1.76-fold increase in CFU/mL compared with the control respectively, no significant difference was observed between strain G1 and G3. The cell density represents the whole cells, while viable cell counts provide the actually viable cells which can work for phloroglucinol production under stress. Therefore the cell viability assays can act as a better measure of cells that are still biologically active during phloroglucinol exposure.

### Effect of GroESL overexpression on phloroglucinol production in shake-flask

**Fig. 4:**
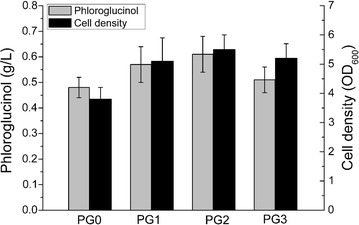
Phloroglucinol production of different recombinant strains in shake flask. Gray bars represent phloroglucinol concentration in the culture broth (g/L); dark bars represent cell density (OD_600_). Data were acquired after each strain was induced with 0.2 mM IPTG at 30 °C for 12 h. Error bars indicate standard deviations (n = 3)

To test the effect of strains with different GroESL expression levels on the production of phloroglucinol, the plasmid pA-phlD/marA/acc and one GroESL overexpression plasmid were transformed into *E. coli* BL21(DE3) to form different recombinant strains (PG1, PG2, PG3). The strain PG0 was used as the control strain. All strains were cultivated in fermentation media under shake-flask conditions. The cell densities and phloroglucinol titers are shown 12 h after induction by 0.2 mM IPTG (Fig. 4). The phloroglucinol titer of strain PG2 reached 0.61 g/L, and was about 1.27-fold to the control strain without GroESL overexpression (0.48 g/L). Therefore, strain PG2 accumulated phloroglucinol much faster than the control strain. In addition, the cell density of PG2 was also higher than the control. Compared with the control, the final phloroglucinol concentration and cell density also increased in strain PG1 and PG3. There are minor differences between the phloroglucinol titers of strain PG1 and PG3 with the values of 0.57 ± 0.07 and 0.51 ± 0.05 g/L, respectively.

These results indicate that the GroESL overexpression leads to a considerable increase in phloroglucinol production, and GroESL expression levels have different effects on strain tolerance and product synthesis. The findings are consistent with the experimental data reported before which concluded that GroESL overexpressing strains using a nisin-inducible expression vector could enhance tolerance compared with the recombinant strains without induction [15]. However, these data are contrary to the findings about the solvent resistance in *E. coli* that no significant difference in tolerance was observed between the high copy inducible and low copy number plasmid [23].

### Fed-batch cultivation of the engineered *E. coli* strain

In order to further evaluate whether the GroESL overexpressing strains could improve phloroglucinol production under fed-batch conditions, the characteristics analyses of the engineered strains were performed in a bioreactor. The strain PG0 was used as the control strain.

**Fig. 5:**
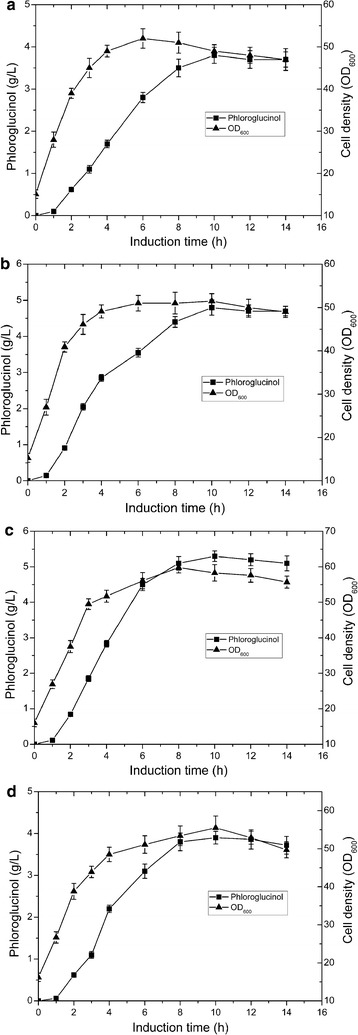
Changes of phloroglucinol concentration (■) and cell density (OD_600_) (▲) over time during the fed-batch cultivation of different engineered *E. coli* strains in a 5-L bioreactor. **a** Strain PG0. **b** Strain PG1. **c** Strain PG2. **d** Strain PG3. Error bars indicate standard deviations (n = 3)

After induction by IPTG, the residual glucose in bioreactor was maintained at about 0.5 g/L to avoid acetate accumulation throughout the fed-batch process, and also ensure adequate substrate supply. Glucose consumption, cell density, and phloroglucinol concentration were monitored during the whole experiment process. The changes for cell density and phloroglucinol concentration of four strains are shown during the fermentation processes (Fig. 5). Compared with the control strain, higher cell densities and phloroglucinol titers were obtained in all GroESL overexpressing strains.

For the recombinant strain PG2, the phloroglucinol titer reached 5.3 ± 0.15 g/L after induction 10 h, corresponding to a 39.5% increase compared with the control strain PG0. A productivity of 0.53 g/L/h was achieved in strain PG2, showing the higher phloroglucinol titer and productivity than previous reported data by microbial synthesis in fermentation broth [7, 9]. The phloroglucinol titers of PG1 and PG3 reached the highest levels after induction 10 h, 4.8 ± 0.21 and 3.9 ± 0.15 g/L, respectively. For all GroESL overexpressing strains, phloroglucinol accumulation hardly increased after induction 10 h even though the viable cells could still be detected, which could be explained by the fact that high concentrations of phloroglucinol can significantly inhibit *E. coli* metabolism. The OD_600_ of strain PG2 reached a maximum of about 59 after induction 8 h, and then the cell density stopped to increase. The cell densities of other two GroESL overexpressing strains also reached maximum after induction 8 h, but the control strain no longer continued to grow after induction 6 h. Moreover, the cell density of control strain was almost lower than strain PG1 and PG2 during the whole fermentation process.

**Fig. 6:**
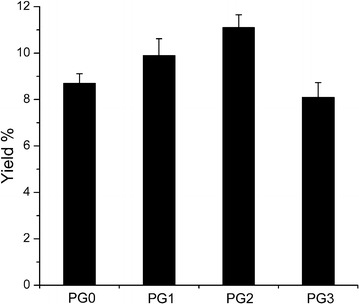
Phloroglucinol yields on glucose during the fed-batch cultivation of strains PG0, PG1, PG2 and PG3 in a 5-L bioreactor. Error bars indicate standard deviations (n = 3)

The yields were calculated by consumed glucose (Fig. 6), and showed the values of 8.7, 9.9, 11.1, and 8.1% in strains PG0, PG1, PG2, PG3, respectively. Strain PG1 and PG2 had higher phloroglucinol yields compared with the control strain PG0, but it was interesting that the yield of strain PG3 was lower than the control strain. These results may be due to the fact that the strain PG3 expressed a large amount of chaperone proteins and consumed more glucose, while the protein expression of strain PG1 and PG2 maintained at an appropriate level.

The results clearly demonstrate that overexpressing *groESL* gene is an effective way to improve phloroglucinol tolerance and production in *E. coli*, and thus modulating the GroESL overexpression at an appropriate level can achieve a better result in production and yield.

## Discussion

Microbial production of various useful chemicals becomes more and more important to our daily life [28]. However, the potential product toxicity to cells is one of the main issues when producing many valuable chemicals by engineered strains from renewable resources [29, 30], because the toxicity can inhibit the product synthesis and cell viability. Product toxicity usually interferes with important metabolic processes and damages cell membrane and cellular macromolecules, thus leading to poor cell viability. Furthermore, stresses on engineered strains can influence metabolic imbalances due to enzyme overexpression against the stress response, which will redistribute the metabolic fluxes and change the intracellular redox state [31]. These impacts will slow down product synthesis and diminish cell viability, and therefore improving product tolerance is an essential aspect for engineered strains. To address these challenges, many metabolic engineering strategies had been used to improve microbial tolerance in order to make chemicals production economically feasible [32–34]. On the other hand, robust cell viability is required for efficient production of chemicals using engineered strains, because the effective cells can lead to high production and productivity via allowing repeated cell recycling. Cell viability is affected by various factors in which cell tolerance to stress is crucial. It has been shown that overexpression of *groESL* gene is an important tool for improving cell viability and tolerance [13, 23]. So far, reported studies mainly focused on increasing solvent resistance [25, 35]. There is no report on increasing phloroglucinol resistance by overexpression of GroESL.

In this study, the GroESL was overexpressed in *E. coli* using different promoters and the effect on phloroglucinol tolerance and production was examined. Overexpressing strains showed better resistance, achieved more viable cells and accumulated higher phloroglucinol titer than the control strain, especially strain PG1 and PG2. This may be due to the fact that GroESL can regulate the synthesis and the activity of σ^32^ which controls the transcriptional level of HSPs and is responsible for *E. coli* cells to respond to a variety of stresses [36]. This modulation may keep the enzyme for phloroglucinol production in a more and longer stabilized state, thus leading to pull the carbon flow towards phloroglucinol. It also means that the overexpressing strain can make the aspects of the stress response system being better controlled than the cell’s natural response to stress. A 27.6% increase in yield was also found in strain PG2 compared with the control strain. However, the yield of glucose to phloroglucinol is 46% in theory, according to glucose consumption based on the following equivalent: 1.5 glucose–phloroglucinol [37]. Therefore, the current yield is still too low and has the potential to be further improved. From an industrial perspective, the related genes activated by overexpressing GroESL should be further analyzed in order to improve the phloroglucinol resistance of *E. coli*, because increasing cell tolerance and viability in high phloroglucinol titers are essential for making a successful industrial process. Recently, the overexpression of GroESL had also been successfully applied to improve cellular robustness and butanol titers of an engineered *Clostridium acetobutylicum* [38].

In SDS-PAGE analysis, different GroESL expression levels depending on promoter strength were found in each strain (Fig. 1). Moreover, the viable cells of recombinant strains with a variety of GroESL expression levels using different promoters were examined under phloroglucinol stress. In this study, the high expression level of GroESL may not be always helpful for cell tolerance, but the appropriate expression level of GroESL may be required for other genes or proteins to properly function. The same results were also found in other study [39], and it is possible that the excessive expression of GroESL protein may cause metabolic burden in host strain and weaken its resistance function. The study proved the importance of metabolic balance in the phloroglucinol artificial biosynthetic pathway, and gave us a guide for the regulation of each protein expression level in order to obtain efficient anabolism. To modulate the expression levels of *groESL* genes had been proven to be important to improve heat and salt tolerance of *Lactococcus lactis*, and protein expression in *E. coli* [15, 40]. Therefore, the specific expression level is an important aspect to improve the tolerance for an effective engineered strain.

The abilities of phloroglucinol production for different recombinant strains were also evaluated by shake-flask and fed-batch fermentations. Compared with the control strain, high phloroglucinol titers were produced by all GroESL overexpressing strains. Although the cell density of strain PG3 was higher than strain PG1, the final phloroglucinol titer of strain PG3 was slightly lower than strain PG1. In general, the changing trend of phloroglucinol titers among recombinant strains was consistent with the number of viable cells measured by plate count method. Hence, the use of cell growth as a sole measure is not sufficient for demonstrating phloroglucinol tolerance, instead of choosing the viable cells assay to examine cell growth. In the future, the viable cell density could be measured with a viable cell monitor (e.g. Aber instruments, UK) in fermentation broth, which seems to be a more reasonable and effective metric for potential capacity of engineered strain [31]. The results show that the appropriate expression level of GroESL can not only improve phloroglucinol production but also impart cellular robustness. In the next stage, the molecular mechanism for phloroglucinol mediated toxicity need be studied for the further application of the GroESL in phloroglucinol synthesis.

## Conclusions

In this study, overexpression of native *groESL* chaperones system was performed to improve phloroglucinol tolerance and production in *E. coli*. The GroESL overexpressing strains not only slightly improved the capacity to produce phloroglucinol on glucose but also increased the cell viability. Then, the GroESL expression levels were further regulated by different promoters, and the highest tolerance and production were observed in the GroESL overexpressing strain under tac promoter. Finally, the strain PG2 accumulated phloroglucinol up to 5.3 g/L after induction 10 h with 39.5% increase compared with the control strain under fed-batch fermentation, demonstrating that overexpression of stress-induced protein GroESL is a promising approach to improve phloroglucinol tolerance and production in *E. coli*. Additionally, the GroESL overexpression appears to improve the cell viability which is demanded for high yields and titers. Although the progress was already obtained in *E. coli* for phloroglucinol production by overexpression of GroESL, increased tolerance and production were only reported under laboratory conditions in these experiments. Therefore, further optimization work need to be done to develop the strain suitable for industrial applications.

## Additional file

**Additional file 1: Table S1.** Primers used in this study.

## References

[1] Yang F, Cao YJ (2012). Biosynthesis of phloroglucinol compounds in microorganisms—review. Appl Microbiol Biotechnol.

[2] Miyake Y, Hiramitsu M (2011). Isolation and extraction of antimicrobial substances against oral bacteria from lemon peel. J Food Sci Tech..

[3] Abdel-Ghany SE, Day I, Heuberger AL, Broeckling CD, Reddy AS (2016). Production of phloroglucinol, a platform chemical, in *Arabidopsis* using a bacterial gene. Sci Rep..

[4] Saharan K, Sarma MVRK, Prakash A, Johri BN, Bisaria VS, Sahai V (2011). Shelf-life enhancement of bio-inoculant formulation by optimizing the trace metals ions in the culture medium for production of DAPG using fluorescent pseudomonad R62. Enzyme Microb Technol.

[5] Weller DM, Landa BB, Mavrodi OV, Schroeder KL, La De, Fuente L, Bankhead SB, Molar RA, Bonsall RF, Mavrodi DV, Thomashow LS (2007). Role of 2,4-diacetylphloroglucinol-producing fluorescent *Pseudomonas* spp. in the defense of plant roots. Plant Biol..

[6] Achkar J, Xian M, Zhao HM, Frost JW (2005). Biosynthesis of phloroglucinol. J Am Chem Soc.

[7] Cao YJ, Jiang XL, Zhang RB, Xian M (2011). Improved phloroglucinol production by metabolically engineered *Escherichia coli*. Appl Microbiol Biotechnol.

[8] Zha W, Rubinpitel SB, Shao Z, Zhao H (2009). Improving cellular malonyl-CoA level in *Escherichia coli* via metabolic engineering. Metab Eng.

[9] Rao GD, Lee JK, Zhao HM (2013). Directed evolution of phloroglucinol synthase PhlD with increased stability for phloroglucinol production. Appl Microbiol Biotechnol.

[10] Nicolaou SA, Gaida SM, Papoutsakis ET (2010). A comparative view of metabolite and substrate stress and tolerance in microbial bioprocessing: from biofuels and chemicals, to biocatalysis and bioremediation. Metab Eng.

[11] Ramos JL, Duque E, Gallegos MT, Godoy P, Ramos-Gonzalez MI, Rojas A, Teran W, Segura A (2002). Mechanisms of solvent tolerance in gram-negative bacteria. Annu Rev Microbiol.

[12] Weber FJ, de Bont JAM (1996). Adaptation mechanisms of microorganisms to the toxic effects of organic solvents on membranes. Biochim Biophys Acta.

[13] Zingaro KA, Papoutsakis ET (2012). Toward a semisynthetic stress response system to engineer microbial solvent tolerance. Mbio..

[14] Alsaker KV, Paredes C, Papoutsakis ET (2010). Metabolite stress and tolerance in the production of biofuels and chemicals: gene-expression-based systems analysis of butanol, butyrate, and acetate stresses in the anaerobe *Clostridium acetobutylicum*. Biotechnol Bioeng.

[15] Desmond C, Fitzgerald GF, Stanton C, Ross RP (2004). Improved stress tolerance of GroESL-overproducing *Lactococcus lactis* and probiotic *Lactobacillus paracasei* NFBC 338. Appl Environ Microb..

[16] Goodarzi H, Bennett BD, Amini S, Reaves ML, Hottes AK, Rabinowitz JD, Tavazoie S (2010). Regulatory and metabolic rewiring during laboratory evolution of ethanol tolerance in *E. coli*. Mol Syst Biol..

[17] Reyes LH, Almario MP, Kao KC (2011). Genomic library screens for genes involved in *n*-butanol tolerance in *Escherichia coli*. PLoS ONE.

[18] Minty JJ, Lesnefsky AA, Lin F, Chen Y, Zaroff TA, Veloso AB, Xie B, McConnell CA, Ward RJ, Schwartz DR, Rouillard JM, Gao Y, Gulari E, Lin XN (2011). Evolution combined with genomic study elucidates genetic bases of isobutanol tolerance in *Escherichia coli*. Microb Cell Fact.

[19] Martin-Galiano AJ, Overweg K, Ferrandiz MJ, Reuter M, Wells JM, Adela G (2005). Transcriptional analysis of the acid tolerance response in *Streptococcus pneumoniae*. Microbiology.

[20] Winkler J, Kao KC (2011). Transcriptional analysis of *Lactobacillus brevis* to *N*-butanol and ferulic acid stress responses. PLoS ONE.

[21] Baneyx F, Mujacic M (2004). Recombinant protein folding and misfolding in *Escherichia coli*. Nat Biotechnol.

[22] Kerner MJ, Naylor DJ, Ishihama Y, Maier T, Chang HC, Stines AP, Georgopoulos C, Frishman D, Hartl FU (2005). Proteome-wide analysis of chaperonin-dependent protein folding in *Escherichia coli*. Cell.

[23] Zingaro KA, Papoutsakis ET (2013). GroESL overexpression imparts *Escherichia coli* tolerance to *i*-, *n*-, and 2-butanol, 1,2,4-butanetriol and ethanol with complex and unpredictable patterns. Metab Eng.

[24] Abdelaal AS, Ageez AM, Abd El-Hadi AA, Abdallah NA (2015). Genetic improvement of *n*-butanol tolerance in *Escherichia coli* by heterologous overexpression of groESL operon from *Clostridium acetobutylicum*. 3. Biotech..

[25] Tomas CA, Welker NE, Papoutsakis ET (2003). Overexpression of groESL in *Clostridium acetobutylicum* results in increased solvent production and tolerance, prolonged metabolism, and changes in the cell’s transcriptional program. Appl Environ Microbiol.

[26] Marc J, Grousseau E, Lombard E, Sinskey AJ, Gorret N, Guillouet SE (2017). Over expression of GroESL in *Cupriavidus necator* for heterotrophic and autotrophic isopropanol production. Metab Eng.

[27] Suo YK, Luo S, Zhang YN, Liao ZP, Wang JF (2017). Enhanced butyric acid tolerance and production by Class I heat shock protein-overproducing *Clostridium tyrobutyricum* ATCC 25755. J Ind Microbiol Biot..

[28] Lee JW, Kim HU, Choi S, Yi J, Lee SY (2011). Microbial production of building block chemicals and polymers. Curr Opin Biotech..

[29] Dunlop MJ (2011). Engineering microbes for tolerance to next-generation biofuels. Biotechnol Biofuels.

[30] Zingaro KA, Nicolaou SA, Papoutsakis ET (2013). Dissecting the assays to assess microbial tolerance to toxic chemicals in bioprocessing. Trends Biotechnol.

[31] Lo TM, Teo WS, Ling H, Chen BB, Kang A, Chang MW (2013). Microbial engineering strategies to improve cell viability for biochemical production. Biotechnol Adv.

[32] Bernal P, Segura A, Ramos JL (2007). Compensatory role of the cis–trans-isomerase and cardiolipin synthase in the membrane fluidity of *Pseudomonas putida* DOT-T1E. Environ Microbiol.

[33] Zhang F, Qian XH, Si HM, Xu GC, Han RZ, Ni Y (2015). Significantly improved solvent tolerance of *Escherichia coli* by global transcription machinery engineering. Microb Cell Fact.

[34] Foo JL, Jensen HM, Dahl RH, George K, Keasling JD, Lee TS, Leong S, Mukhopadhyay A (2014). Improving microbial biogasoline production in *Escherichia coli* using tolerance engineering. MBio..

[35] Tomas CA, Beamish J, Papoutsakis ET (2004). Transcriptional analysis of butanol stress and tolerance in *Clostridium acetobutylicum*. J Bacteriol.

[36] Arsène F, Tomoyasu T, Bukau B (2000). The heat shock response of *Escherichia coli*. Int J Food Microbiol.

[37] Zha W, Rubin-Pitel SB, Zhao H (2008). Exploiting genetic diversity by directed evolution: molecular breeding of type III polyketide synthases improves productivity. Mol BioSyst.

[38] Liao ZP, Zhang YN, Luo S, Suo YK, Zhang SZ, Wang JF (2017). Improving cellular robustness and butanol titers of *Clostridium acetobutylicum* ATCC824 by introducing heat shock proteins from an extremophilic bacterium. J Biotechnol.

[39] Tomoyasu T, Mogk A, Langen H, Goloubinoff P, Bukau B (2001). Genetic dissection of the roles of chaperones and proteases in protein folding and degradation in the *Escherichia coli* cytosol. Mol Microbiol.

[40] de Marco A (2007). Protocol for preparing proteins with improved solubility by co-expressing with molecular chaperones in *Escherichia coli*. Nat Protoc.

