# Supplementary material for: Improving phloroglucinol tolerance and production in Escherichia coli by GroESL overexpression
Source: Microb Cell Fact. 2017 Dec 19;16:227. doi: 10.1186/s12934-017-0839-x (PMC5735909; doi:10.1186/s12934-017-0839-x)
Supplement: Supplementary file 1 — Additional file 1: Table S1. Primers used in this study. [file 12934_2017_839_MOESM1_ESM.docx]

Additional file 1: Table S1 Primers used in this study

| **Primers** | **Sequence** |
| --- | --- |
| phlDmarA-F | ATAAGAATGCGGCCGCTCGATCTCGATCCCGCGAAAT |
| phlDmarA-R | ATCGCTTAAGCTAGCTGTTGTAATGATTTAATGGATG |
| groESL-F_BglII | GAAGATCTGGGTTGATGTCCGATTGCGCCCAAA |
| groESL-R_EcoRI | GGAATTCTTACATCATGCCGCCCATGCCACCCAT |
| T7-groESL-F_NcoI | CATGCCATGGGCAATATTCGTCCATTGCATGATCGCGT |
| T7-groESL-R_EcoRI | GGAATTCTTACATCATGCCGCCCATGCCACCCAT |
| tac-groESL-F_BglII | GATCTCGATCCCGCGAAATTGACAATTAATCATCGGCTCGTATAATGTGTGGAATTGTGAGCGGATAACAATTCCCCT |
| tac-groESL-R_XbaI | CTAGAGGGGAATTGTTATCCGCTCACAATTCCACACATTATACGAGCCGATGATTAATTGTCAATTTCGCGGGATCGA |
